# Factors associated with patients self-reported adherence to prescribed physical activity in routine primary health care

**DOI:** 10.1186/1471-2296-11-38

**Published:** 2010-05-19

**Authors:** Matti E Leijon, Preben Bendtsen, Agneta Ståhle, Kerstin Ekberg, Karin Festin, Per Nilsen

**Affiliations:** 1 Center for Primary Health Care Research, Lund University/Region Skåne, Malmö, Sweden; 2 Department of Medical and Health Sciences, Division of Community Medicine, Social Medicine and Public Health Science, Linköping University, Linköping, Sweden; 3 Department of Neurobiology, Health Care Sciences and Society, Division of Physiotherapy, Karolinska Institutet, Stockholm, Sweden; 4 Department of Medical and Health Sciences, Division of Community Medicine, National Centre for Work and Rehabilitation, Linköping University, Linköping, Sweden

## Abstract

**Background:**

Written prescriptions of physical activity have increased in popularity. Such schemes have mostly been evaluated in terms of efficacy in clinical trials. This study reports on a physical activity prescription referral scheme implemented in routine primary health care (PHC) in Sweden. The aim of this study was to evaluate patients' self-reported adherence to physical activity prescriptions at 3 and 12 months and to analyse different characteristics associated with adherence to these prescriptions.

**Methods:**

Prospective prescription data were obtained for the general population in 37 of 42 PHC centres in Östergötland County, during 2004. The study population consisted of 3300.

**Results:**

The average adherence rate to the prescribed activity was 56% at 3 months and 50% at 12 months. In the multiple logistic regression models, higher adherence was associated with higher activity level at baseline and with prescriptions including home-based activities.

**Conclusions:**

Prescription from ordinary PHC staff yielded adherence in half of the patients in this PAR scheme follow-up.

## Background

Written prescriptions of physical activity, in Sweden commonly referred to as physical activity referral (PAR) schemes [1], have increased in popularity in recent years [1-7]. PAR schemes were initially developed in the UK [4] and were later introduced more broadly in Sweden in 2001 by the National Institute of Public Health in a national campaign called "Sweden on the move" [5,8]. Swedish PAR schemes typically entail primary health care (PHC) providers issuing a formal written physical activity prescription for home-based activities, such as walking, or facility-based activities organized by different physical activity organizations in the community [1,5,8,9].

So far PAR schemes have mostly been studied in terms of efficacy, employing randomized controlled trial study designs and researcher-assisted study protocols [6]. Establishing efficacy is usually an important first step before widespread dissemination and implementation of new interventions. The effectiveness of PARs has been questioned by some researchers [4,10,11], although the efficacy has been supported by randomized controlled trials presented in a number of reviews in recent years [6,7,12,13]. However, the enhanced internal validity accomplished in such research is often gained at the expense of external validity since the study conditions tend to be far removed from routine practice. Indeed, interventions in many health fields that have been found to be successful in efficacy studies have proved impractical to implement in applied settings that have limited time, few resources, and many competing demands [14,15]. There is a paucity of pragmatic PAR studies conducted in routine practice that involve more heterogeneous populations [15]. Furthermore, many trials have measured physical activity by using instruments that are scored on a scale that does not easily convert to a pragmatic counselling message, thus restricting their clinical usefulness [16].

It has been suggested that adherence to PAR should be evaluated by simply asking the patient about the degree of adherence to the prescription, which is a pragmatic and realistic approach from a routine practice perspective. This approach is easy to incorporate into a real-life setting, being simple to use, inexpensive, and not time-consuming [17]. Adherence has been defined by the WHO [18] as "the extent to which a person's behaviour, taking medication, following a diet, and/or executing lifestyle changes, corresponds with agreed recommendations from a health care provider". There is no gold standard for assessment of adherence in general and no validated self-reporting question exists to measure adherence to physical activity interventions [18]. Few physical activity studies have examined adherence as a primary outcome variable [19].

The present study addresses a knowledge gap with regards to effectiveness of a Swedish PAR scheme implemented in routine PHC. We aimed to assess the effectiveness of a Swedish PAR scheme in routine PHC by evaluate patients' self-reported adherence to PARs at 3 and 12 months and to analyse different characteristics associated with adherence to these prescriptions.

## Methods

### Study setting

The study was conducted in primary health care (PHC) in the county of Östergötland, Sweden, in the year 2004. This county of 416,000 inhabitants is the fourth largest county in Sweden and includes two large cities (> 120,000 inhabitants) and 11 smaller, more rural municipalities. At the time of the study, the County Council encompassed three hospitals and 42 PHC centres, of which four were privately owned and 38 were managed by the County Council.

All PHC centres in Östergötland have a specified catchment area and/or a listed population (ranging from 3,700 to 20,700 patients per unit). The PHC centres usually include different health care professionals, i.e. physicians, nurses, physiotherapists, occupational therapists, dieticians, and behavioural scientists. The number of staff in the PHC centres ranged from 10 to 80, with the number of physicians ranging from 2 to 12 and nurses from 8 to 35 (as of 31 December 2004).

The PAR scheme in Östergötland was built on structures developed over a number of years, based on collaborations between local physical activity organizations and PHC centres. This included the development of a widely used locally adapted prescription form, information materials, and knowledge exchange among the actors involved.

At the end of 2003, 80% of the PHC centres in the region worked with PARs to some extent and had established a supportive community-based structure to assist patients to gain access to various local activities[9].

Ethical approval was not required for this follow-up as the data collection was part of the routine health care system.

### The prescription procedure

The prescription procedure was intended to be patient-centred, and to take into consideration the patient's current activity level, activity history, capacity, motivation, and interests. Persons eligible to receive PARs were all ordinary PHC patients whom the regular staff believed would benefit from increased physical activity. Swedish PARs consist of activities that are home-based and/or self-monitored, such as walking, jogging or cycling, and facility-based activities organised by different physical activity organisations in the community. The patients either had a sedentary lifestyle or a diagnosis that indicated that increased physical activity could be beneficial, e.g. high blood pressure, diabetes, and/or musculoskeletal disorders.

The patient was provided with a written PAR and a copy was kept in the patient's medical record. If the activity prescribed was facility-based (e.g. group gymnastics, aerobics, water aerobics, weight and circuit training.), a copy was also sent to the PARs coordinator in the relevant physical activity organization, who then contacted the patient by telephone or letter. The patients paid the normal fee to the organization they attended. The physical activity organization also made a phone call after 5 weeks to verify if the patient had attended the suggested group activity. The purpose of the phone call was threefold: (1) to guide and motivate potential drop-out patients to participate in other activities; (2) to give other patients/participants the opportunity to attend instead of drop-out patients; (3) and to gather information about drop-outs for feedback to the PHC centres. Patients who were prescribed home-based activities, such as walking, did not receive this phone call.

### Study population

Patients were recruited prospectively from 37 of the 42 PHC centres in the county. Of the five centres that did not participate, two were public PHC centres that did not work with PARs and three private PHC centres declined to participate due to lack of time. A 3-month follow-up on patients issued physical activity on prescription was conducted by 36 centres and a 12-month follow-up was conducted by 27 centres. The main reasons for non-participation in follow-ups by PHC centres were lack of time or shortage of staff.

### Data collection

All prescription forms were registered by the PARs coordinator in each unit in a Microsoft Excel spreadsheet, which was sent to the first author three times a year.

Follow-up measures were performed by PHC personnel. Three different methods were used to collect the questionnaire data: telephone interview, postal questionnaire, and/or questionnaire provided during the patient's normal return visit. At the 3-month follow-up, 74% of the patients were contacted by telephone, 14% by postal questionnaire, and 12% answered the follow-up questions during a return visit. The 12-month follow-up showed a similar pattern with 68% contacted by telephone, 21% by postal questionnaire, and 11% during a return visit.

### Baseline measures

The prescription form used to collect the baseline data included patient data such as age, sex, address, telephone number, and information about the prescriber's profession.

Patients were asked to state the number of days in the previous week (7-day recall) "with at least a total of 30 minutes of physical activity that made you warm, e.g. brisk walking, gardening, heavy housework, cycling and/or swimming". This short and simple question was used for practical reasons, and was based on the current physical activity recommendation in Sweden. In the analysis, the patients' self-reported physical activity was classified into four groups: (1) regularly active (those who reported 5-7 days of 30 minutes of moderately intense physical activity); (2) moderately active (3-4 days); (3) somewhat active (1-2 days); and (4) inactive (0 days). Data including additional baseline data and data regarding physical activity level before and after the intervention are presented elsewhere[9].

Reasons for receiving PARs were registered on the prescription form by selecting one or more of seven predefined options including sedentary lifestyle. The disease-specific options were musculoskeletal disorders, overweight (body mass index > 25), diabetes, high blood pressure, high blood cholesterol, and mental ill-health. The "other PARs reasons" included asthma and chronic pulmonary disease. Patients issued prescriptions for more than one reason were categorized as "combination of reasons/diagnoses".

The activities could either be home-based (free-living or lifestyle activities such as walking) or structured facility-based provided by a local physical activity organization. Patients who were issued home-based activities and structured facility-based activities were classified into a combination category.

### Follow-up measures at 3 and 12 months

The patients' self-reported adherence to the issued activity was measured by asking the patient the question "have you adhered to your physical activity prescription?" The respondent selected one of three alternatives: (1) "I adhered to the prescription"; (2) "I'm active but in another activity than the prescribed activity"; (3) "I do not follow my prescription". Results are presented as (1) adhered, (2) partly adhered, and (3) non-adhered. Follow-ups also included the same physical activity question, and the patients were asked to state their current physical activity, data presented elsewhere [1].

### Statistical analyses

In the descriptive analyses, differences between proportions were analysed with the non-parametric chi-square test.

Univariate and multiple logistic regression analyses were applied to identify possible associations between self-reported adherence, and sex, age, activity level at baseline, referred activity type, referral practitioner, and reason for prescription of physical activity. Separate analyses were done for the 3- and 12-month follow-ups. As the aim of the study was to analyse adherence, patients reporting part adherence were excluded from these analyses, e.g. outcome measure was adhered vs. not adhered. All variables with a *p*-value < 0.2 in the univariate logistic analyses were included in the multiple logistic regression analyses. In the two final multiple models, all possible two- and three-way interaction terms were tested.

Statistical significance was set at *p *< 0.05 and the confidence interval was 95%. SPSS (release 15.0) software was used for all analyses.

## Results

### Participation rates

There were 2753 patients, from 36 PHC centres, available for the 3-month follow-up. Since nine PHC centres decided not to participate in the 12-month follow-up, 1992 patients, from 27 PHC centres, available for this follow-up. The external patient drop-out was very low and resulted in a follow-up rate of 98% (2704 of 2753) at the 3-month follow-up and 99% (1965 of 1992) at the 12-month follow-up.

Only patients who responded to the question on adherence were included in the analyses, leaving 2612 patients for the 3-month follow-up and 1907 patients at the 12-month follow up. The internal drop-out rate ranged from 0% (age) to 11% (activity level at baseline) for the questions analysed.

The patient characteristics did not differ significantly between baseline (*n *= 3300) and the 3-month follow-up (*n *= 2753) or 12-month follow-up (*n *= 1992), for age, sex, activity level at baseline, referred activity type, referral practitioner or reasons for prescription.

### Patient characteristics and adherence to PARs

The mean age of those included in this study was 54 years (SD 14.2). Two out of three (66.6%) patients were female. As shown in Table 1, more than half (56%) of the patients reported adherence to the prescribed activity at the 3-month follow-up. Almost one-fifth (18%) of the patients were active but in another activity than the prescribed one (partly adhered). At the 12-month follow-up, half (50%) of the patients reported adherence and 21% reported that they partly adhered to the prescription. There were no statistically significant differences between females and males in adherence at 3 or 12 months (*p *= 0.467 and *p *= 0.812, respectively).

**Table 1:** Adherence to prescribed physical activity, descriptive analysis: percentage of patients who reported adherence, part adherence, and non-adherence to PARs after 3 and 12 months

	3-month follow-up					12-month follow-up				
	
	*n*	Adhered (%)	Partly adhered (%)	Non-adhered (%)	*p*-value	*n*	Adhered (%)	Partly adhered (%)	Non-adhered (%)	*p*-value
**Total**	2611	56	18	26		1846	50	21	30	
**Sex**					0.467					0.812
Female	1740	57	17	26		1223	49	21	29	
Male	871	55	19	26		623	50	20	30	
**Age (groups)**					0.218					0.037
18-29	117	48	20	33		76	40	18	42	
30-44	545	53	19	28		383	44	25	31	
45-64	1337	58	18	25		938	52	20	28	
> 65	613	58	18	25		450	51	20	29	
**Activity level at baseline (7-day recall)**					< 0.001					< 0.001
0 days	841	49	14	37		614	40	19	41	
1-2 days	675	59	18	23		505	52	20	28	
3-4 days	336	67	20	13		240	58	21	21	
5-7 days	475	60	21	19		320	57	26	17	
**Activity type**					< 0.001					< 0.001
Home-based activity	940	71	10	20		710	62	11	27	
Facility-based activity	1206	44	25	32		828	35	31	34	
Combination of home-based and facility-based activity	442	61	17	22		301	58	18	24	
**Referral practitioner**					< 0.001					< 0.001
Physician	974	51	21	28		737	46	25	29	
Nurse	807	61	12	27		573	55	13	31	
Physiotherapist	380	56	23	21		268	42	27	31	
Other	395	63	18	20		268	54	19	28	
**Reasons for prescription**					0.005					< 0.001
Sedentary	133	54	15	31		100	44	17	39	
Musculoskeletal	552	53	21	26		359	42	32	26	
Overweight	285	53	19	28		180	41	25	34	
Diabetes	195	68	14	18		196	57	17	27	
High blood pressure	187	64	15	21		141	64	11	26	
Cholesterol	20	55	15	30		18	67	28	6	
Mental ill health	102	53	24	24		63	43	22	35	
Other PAR reasons	63	57	24	19		43	61	19	21	
Combination of reasons/diagnosis	981	55	17	28		747	51	18	32	

Higher adherence was associated with increased age (12 months follow-up only), higher activity level at baseline, home-based activities, prescriptions issued by professional groups other than physicians at 3 months and physicians and physiotherapists at 12 months. Adherence was higher among patients issued PARs due to prescription reasons or diagnoses like diabetes and high blood pressure. The descriptive analyses also found that approximately half (52%) of those reporting adherence to PARs also increased their physical activity level between baseline and follow-up (at the 3- and 12-month follow-up).

The univariate logistic regression analyses (not shown) indicated no adherence differences according to sex at follow-ups. The odds ratios according to age groups showed significance at 12 months (*p *= 0.043) resulting in higher adherence among the older age groups. A tendency to higher adherence among older patients was also found at 3 months (*p *= 0.058). Higher adherence was significantly associated with higher activity level at baseline (*p *< 0.001), home-based activities (*p *< 0.001), being referred by a nurse or "other practitioner" (*p *> 0.001) and also for prescription reasons (*p *= 0.005 at 3 months and *p *< 0.001 at 12 months); higher adherence was found among patients with high blood pressure, diabetes and high cholesterol level.

As shown in Table 2, in the multiple logistic regression model higher adherence was also associated with higher activity level at baseline (*p *< 0.001). Patients referred to structured facility-based activities showed a lower adherence compared to those referred to a combination of home-based and facility-based activities (*p *< 0.001). Those two associations were true at both 3 and 12 months. At 3 months, an apparent association between adherence and referral practitioner was indicated, showing higher adherence among physiotherapists than among physicians. However, this association was caused by an imbalance in the type of activity prescribed by the practitioners (*p *< 0.001).

**Table 2:** Adherence to prescribed physical activity, multiple logistic regression analysis: odds ratio for adherence to physical activity prescriptions in routine primary health care

	3 months			12 months		
	
	*p*-value	Odds ratio	95% CI	*p*-value	Odds ratio	95% CI
**Activity level at baseline (7-day recall)**	< 0.001			< 0.001		
0 days		1.00			1.00	
1-2 days		1.83	1.42-2.35		1.75	1.33-2.310
3-4 days		3.92	2.67-5.77		2.69	1.84-3.92
5-7 days		2.14	1.60-2.87		3.38	2.36-4.84
**Activity type**	< 0.001			< 0.001		
Home-based activity		1.88	1.15-3.07		1.06	0.75-1.50
Facility-based activity		0.49	0.32-0.76		0.47	0.33-0.66
Combination of home-based and facility-based activity		1.00			1.00	
**Referral practitioner**	0.254			(0.829)^a^		
Physician		1.00				
Nurse		1.08	0.61-1.93			
Other		1.64	0.57-4.71			
Physiotherapist		2.34	0.96-5.73			
Activity type * Referral practitioner	< 0.001					
Home-based activity * Nurse		0.78	0.38-1.60			
Home-based activity *Other		0.27	0.08-0.92			
Home-based activity * Physiotherapist		0.39	0.14-1.11			
Facility-based activity * Nurse		0.87	0.44-1.72			
Facility-based activity * Other		1.67	0.54-5.19			
Facility-based activity * Physiotherapist		0.84	0.31-2.30			
Combination of home-based and facility-based activity * Physician		1.00				

Multiple logistic regression analyses, adherence vs. non-adherence at 3 months (*n *= 1860) and 12 months (*n *= 1320).

^a^Not included in the model.

Physiotherapists prescribed home-based activities for their patients much less frequently than physicians (21% vs. 41%). Home-based activities had higher adherence than facility-based activities. Furthermore, the few patients referred by a physiotherapist to home-based activities (*n *= 44), showed lower adherence compared to patients referred by physicians (67% vs. 80%). The associations between adherence and age and reasons for prescription were no longer significant after the variables in Table 2 were included, and were therefore excluded in the final models.

## Discussion

This study aimed to evaluate self-reported adherence to physical activity prescriptions issued in everyday PHC at 3 and 12 months and to analyse the characteristics associated with this adherence. Patients were prospectively recruited by regular staff in routine PHC. We measured adherence using a very simple question of whether a patient adhered to the prescribed activity or not. This question is pragmatic and natural to use in clinical practice, but it has not been scientifically validated. There is an obvious risk of recall or social desirability bias with the question we used, but experienced health care professionals have expressed that they believe that patients generally report adherence truthfully and to the best of their ability. While self-reports always carry a potential risk of bias, including social desirability [20], self-reporting tools have generally been found to be accurate and reliable when compared to objective quantification of physical activity through monitoring or directly measured energy expenditure [16,21,22]. There is no gold standard self-reporting measure of adherence to physical activity prescriptions or physical activity levels [17,18]. Many traditional instruments have shortcomings from a clinical perspective. Physical activity levels are often scored on scales that are not easily converted into a counselling message [16,21,22]. It can also be difficult to assess small but clinically significant changes in physical activity levels in a practice situation. Problems with these instruments underscore the challenge of translating research findings into clinical practice and achieving more widespread implementation of PAR schemes [17].

The overall adherence rates seen in this study were relatively high, with 56% of the patients adhering to the prescription at 3 months and 50% at 12 months. However, these results are similar to a previous Swedish PAR study, which reported 53% adherence at 6-month follow-up [5]. This can also be compared with medication adherence in long-term treatment of chronic illness, which averages 50% in developed countries [18].

We found that being physically inactive at baseline was associated with lower adherence. This finding is consistent with previous research, as shown in a review from 2005 [6], which concluded that exercise referral schemes appear to increase physical activity levels in those not sedentary but already slightly active. It would seem that those who are at least slightly active have established a habit of engaging in physical activity, even though the habit may be relatively weak, whereas those who are inactive experience more difficulties in translating motivation and behavioural intentions into actual behaviour change. This suggests that a prescription may not be a sufficiently effective intervention for lowering the threshold for initiating a new behaviour. Instead, some form of personal counselling or the use of some type of motivational technique may be required for many who are physically inactive in order to achieve the desired behavioural change. We chose to include only those adhering to the prescription in our analyses even though those who became physically active in an activity *other *than that prescribed could also be considered positive responders to the PAR intervention. Certainly, increased physical activity in general is a desired outcome. This also raises the question if the PAR intervention in these cases was not well tailored to the specific individual.

Another key finding was that home-based activities were associated with higher adherence than facility-based activities. Although there is insufficient evidence to conclude which types of physical activity are most effective to increase physical activity levels [13], it is likely that relatively simple home-based activities can more easily become habits than more complex behaviours. Activities like walking, jogging or cycling can easily be incorporated into routine daily life, whereas facility-based activities typically require more intentional effort and planning [23]. Differences in adherence between activity types can also be attributed to different preferences and personal characteristics of the participants [19].

Facility-based activities usually require higher intensity than home-based activities such as walking. However, research findings are somewhat inconsistent concerning the relationship between adherence and the frequency and intensity of the issued activity [6,13,19]. Studies from the US have suggested that home-based activities increase levels of moderate physical activity, while facility-based activities might achieve greater improvement in levels of vigorous activity [6]. However, these characteristics and relationships are not yet well understood. It is clearly not a question of "either-or" but rather "both-and", in the sense that home-based and facility-based activities should be viewed as complementary approaches in the promotion of physical activity. Those who are just beginning an activity program may benefit most from some features of facility-based activities, such as individualized instruction and support. Home-based activities, on the other hand, clearly offer increased flexibility, which may be essential for individuals with time or transportation limitations [19].

There was a strong correlation between adherence and increased physical activity level. Three out of five (61%) among those adhering to PARs also increased their self-reported physical activity level between baseline and 12-month follow-up. Increased long-term physical activity cannot be achieved without a certain degree of adherence, which suggests that adherence could be measured as a simple proxy for changes in physical activity level in PARs interventions. These findings confirm the notion that self-reported adherence can be a suitable measure for follow-up at return visits and be a complement to questions concerning physical activity levels [17].

Adherence rates differed between professional groups in the descriptive analysis, with lower adherence for prescriptions issued by physicians. This is in accordance with previous findings [24]. However, differences between professions disappeared in our multiple model, meaning that there was no "profession effect" in this study, when taking the interaction between profession and referred activity type into consideration.

In the univariate logistic analyses, there was higher adherence related to increased age at 12 months. This is consistent with findings in previous studies [4,24], which have also found that patients with certain referral conditions (e.g. myocardial infarction) demonstrate much higher (even doubled) adherence rates than other referral conditions (e.g. mental illness). We also found higher adherence rates associated with age and certain PARs reasons in the descriptive and univariate analyses. However, in the multiple regression model, there were no differences in adherence related to the sex or age of the patient or to the profession of the referral practitioner and the prescription reason/diagnoses, indicating that other factors are more important when predicting adherence.

This study has weaknesses and strengths. As mentioned previously, we relied on self-reports and used a simple adherence question. However, this limitation should be balanced against the study's strengths. We included a large number of patients in a routine care setting which made it possible to do statistically sound sub-group analyses. We also believe the study's external validity is favourable, meaning that many results can be generalized to other populations and settings. It is difficult to achieve a high degree of both internal and external validity in the same study. This study was highly pragmatic, with feasibility and the use of simple questions and procedures a necessity.

Increased physical activity is an important public health objective and effective methods to promote physical activity are needed. It is often challenging to communicate research findings into clinical practice and even more so to introduce new methods. The usage or implementation of complicated methods or instruments related to preventive work in everyday practice may be one of the reasons for the failure of translating effective clinical and community-level services into routine practice, i.e. the so-called translation gap [25,26].

## Conclusions

Prescription from ordinary PHC staff yielded adherence in half of the patients in this PAR scheme follow-up. Patients' activity level at baseline (being at least somewhat physically active) and being issued home-based activities were associated with higher adherence at both 3 and 12 months.

## Abbreviations

PAR: Physical activity referral; PHC: Primary health care

## Competing interests

The authors declare that they have no competing interests.

Funding: County Council of Östergötland

## Authors' contributions

MEL participated in the design of the study, collected data and conducted initial data analyses, drafted and revised the manuscript. PN, PB and AS contributed to interpretation of the data and revision of the manuscript. KE participated in the design of the study and revised the manuscript. KF performed data analyses, contributed to interpretation of the data and revision of the manuscript. All authors read and approved the final manuscript.

## Pre-publication history

The pre-publication history for this paper can be accessed here:

http://www.biomedcentral.com/1471-2296/11/38/prepub
